# Socioeconomic status, education, and aortic stiffness progression over 5 years: the Whitehall II prospective cohort study

**DOI:** 10.1097/HJH.0000000000001057

**Published:** 2016-09-01

**Authors:** Xavier Trudel, Martin J. Shipley, Carmel M. McEniery, Ian B. Wilkinson, Eric J. Brunner

**Affiliations:** a Axe Santé des populations et pratiques optimales en santé, Hôpital St-Sacrement, CHU de Québec, Québec City, Québec, Canada; b Epidemiology & Public Health, University College London, London; c Clinical Pharmacology Unit, University of Cambridge, Addenbrooke's Hospital, Cambridge, UK

**Keywords:** aortic stiffness, prospective cohort study, pulse wave velocity, socioeconomic status

## Abstract

**Objective:**

The inverse association between socioeconomic status (SES) and cardiovascular disease (CVD) risk is well documented. Aortic stiffness assessed by aortic pulse wave velocity (PWV) is a strong predictor of CVD events. However, no previous study has examined the effect of SES on arterial stiffening over time. The present study examines this association, using several measures of SES, and attained education level in a large ageing cohort of British men and women.

**Methods:**

Participants were drawn from the Whitehall II study. The sample was composed of 3836 men and 1406 women who attended the 2008–2009 clinical examination (mean age = 65.5 years). Aortic PWV was measured in 2008–2009 and in 2012–2013 by applanation tonometry. A total of 3484 participants provided PWV measurements on both occasions. The mean difference in 5-year PWV change was examined according to household income, education, employment grade, and father's social class, using linear mixed models.

**Results:**

PWV increase [mean: confidence interval (m/s)] over 5 years was higher among participants with lower employment grade (0.38: 0.11–0.65), household income (0.58, 95%: 0.32–0.85), and education (0.30: 0.01, 0.58), after adjusting for sociodemographic variables, BMI, alcohol consumption, smoking, and other cardiovascular risk factors, namely SBP, mean arterial pressure, heart rate, cholesterol, diabetes, and antihypertensive use.

**Conclusion:**

The present study supports the presence of robust socioeconomic disparities in aortic stiffness progression. Our findings suggest that arterial aging could be an important pathophysiological pathway explaining the impact of lower SES on CVD risk.

## INTRODUCTION

In industrialized countries, cardiovascular diseases (CVD) are the leading causes of mortality [1] and generate important hospitalization costs [2]. A substantial body of evidence has demonstrated that low socioeconomic status (SES) is associated with cardiovascular mortality and morbidity [3]. The socioeconomic gap in CVD incidence has recently widened [4–6] and persists at older age [7].

Aortic pulse wave velocity (PWV) is a measure of the intrinsic stiffness of the aortic wall and a novel surrogate marker of CVD risk. Recent meta-analyses have reported a 15% increased CVD risk for each unit increase in PWV (1 m/s) [8] and an improved CVD risk prediction in different subgroups [9]. Aortic stiffness might be an important predictive summary measure that captures the effect of long-term exposure to a low SES over the life course on vascular ageing.

There is sparse evidence to date on the relation between SES and arterial stiffness, and none examining progression. Ethnicity, low education, family income, and neighborhood deprivation were associated with higher PWV in a small sample of American adolescents [10]. Less educated male Japanese civil servants have been shown to have higher brachial–ankle PWV [11]. Accelerated progression of carotid intima–media thickness, capturing a different aspect of vascular damage and subclinical disease has been linked to lower SES [12–15]; however, carotid intima–media thickness and aortic PWV are at best weakly correlated [16]. There is thus a need for a longitudinal examination of the effect of SES on arterial stiffening over time.

The present study aims to examine the association between SES and PWV progression, using several measures of SES, and attained education level in a large ageing cohort of British men and women.

## METHODS

### Study sample

The Whitehall II study is a longitudinal study of 10 308 male and female civil servants (initially aged 35–55 years) based in London and set up in 1985. The civil service refers to branches of public service concerned with all governmental administrative functions. The baseline response rate was 73%. Details on this cohort of white-collar workers have been published [17]. Participants have been followed with clinical examination every 4–5 years and with questionnaires every 2–3 years up to 2015. The present study sample included 5242 participants who underwent PWV measurement at the 2008–2009 (*n* = 4379) or 2012–2013 (*n* = 4347) assessments, using the same protocol. A total of 3484 participants provided PWV measurements on both occasions.

### Data collection

#### Socioeconomic status and education

Three indicators were used to measure SES: father's social class, employment grade, and household income. In addition, participants reported their highest level of educational attainment.

Father's social class is a frequently used indicator of SES in childhood [18]. It was assessed at the first survey (1985–1988) with the question ‘What is/was your father's main job, what kind of work does/did he do in it’. This was coded based on the Registrar General's occupational classification, one of the main scales used in Britain to measure social class based on occupational status. Father's social class was grouped in a six-level hierarchy I, II, IIINM, IIIM, IV, and V.

Employment grade was measured using current or last known employment grade. The civil service identifies 12 nonindustrial grades: clerical assistant, clerical officer, executive officer, higher executive officer, senior executive officer, and seven ‘unified grades’ of administrator. Other professional and technical staff were assigned to these grades on the basis of salary. Unified grades 1–6 were combined into one group and the bottom two clerical grades into another, producing six categories.

Annual household income in 2008–2009 included the ‘total annual household income from any source, including personal income’. Categories were £100 000 or more, £70 000–99 999, £50 000–69 999, £35 000–49 999, £25 000–34 999, £20 000–24 999, £15 000–19 999, £10 000–14 999, <£9999.

Education was assessed as the highest qualification attained while in full-time education. It was grouped into five categories: No academic qualification, ordinary level, advanced level, BA/BSc, and higher degrees. Ordinary level is the basic level of the General Certificate of Education, whereas the advanced level is a higher and more in depth qualification, usually required for university admission.

A number of participants with missing data on SES and education variables were further excluded in the corresponding analyses (*N* = 1666, 22, 696, and 207 for father's social class, employment grade, household income and education, respectively).

#### Aortic pulse wave velocity

PWV was assessed between the carotid and femoral sites using applanation tonometry (SphygmoCor, Atcor Medical, Sydney, New South Wales, Australia). Path length was determined with a tape measure by subtracting the carotid-sternal notch distance from the femoral-sternal notch distance. In each participant, PWV was measured twice, and if the difference in velocity between the two measurements was larger than 0.5 m/s, a third measurement was taken. The average of all of the measurements was used in the analyses. PWV measurements were repeated within 30 days in 125 participants in 2008–2009 and 114 participants in 2012–2013 to assess short-term reproducibility. Median intraindividual difference in PWV was respectively 0.83 m/s (interquartile range 0.43–1.40) and 0.89 m/s (interquartile range 0.41–1.47).

#### Covariates

Ethnicity was classified as white/nonwhite. Weight, height, and waist circumference were measured according to standard protocols [17]. Smoking status and alcohol intake (yes/no) were collected by questionnaire. Resting heart rate was measured via ECG with participants in the supine position. SBP and DBP were measured twice after 5 min of rest using OMRON HEM 907 (OMRON Healthcare UK Ltd, Milton Keynes, UK) automated monitors [19,20]. The average of SBP was used. From the supine SBP and DBP, mean arterial pressure (MAP) in millimeters of mercury was calculated as follows: DBP + 0.33 (SBP − DBP). Prevalent diabetes mellitus was determined by self-report of doctor diagnosis and/or medication or oral glucose tolerance test. Total cholesterol and high-density lipoprotein cholesterol were measured using automated enzymatic colorimetric methods. Participants taking antihypertensive medication were identified through a questionnaire item on current medication.

### Analyses

#### Slope index of inequality

A slope index of inequality (SII) was computed for each socioeconomic indicator [21]. Individuals in each category were assigned a value equivalent to the proportion of the population with a higher SES than the midpoint of that category. For example, if the highest and next highest employment grade categories include 10 and 20% of the population, respectively, the range of the individuals in the highest category would be from 0 to 0.1 giving a median score of 0.05, which would be assigned to all individuals in this category. Similarly, those in the next highest category would be assigned a score of 0.2 (0.1 +  (0.2/2)), and so on, according to the cumulative range of this category [21]. These scores were then fitted as continuous explanatory variables and the coefficient represented the absolute difference in mean PWV between the lowest (score 1) to the highest level (score 0) of the SES indicator. The strength of the SII is its ability to provide a single summary measure of health disparity, including direction and magnitude, using all data [22].

A SII was computed for each socioeconomic indicator [21]. Each indicator was converted into sex-specific scores; individuals in each category were assigned a value equivalent to the proportion of the population with a higher SES than the midpoint of that category. These scores were then fitted as continuous explanatory variables and the coefficient represented the absolute difference in mean PWV between the highest (score 0) to the lowest level (score 1) of the SES indicator. These indexes were used to examine the cross-sectional and longitudinal association between SES and PWV.

#### Mixed models

Linear mixed models were used to measure the effect of SES on baseline PWV (2008–2009) and PWV longitudinal change between 2008–2009 and 2012–2013. These models use all available data over the follow-up, handle differences in length of follow-up, and account for correlation between repeated measures on the same individual. The linear mixed models included a term for time (individual follow-up in years divided by five, to yield effects of change over 5 years). The main effect estimates the effect of SES on PWV at baseline (2008–2009), whereas the SES × time interaction term estimates the mean difference in the 5-year changes in PWV. The effect of SES on PWV was estimated with categorical SES indicators and with the slope indexes of inequality. For clarity purposes, each SES indicator was regrouped into three categories. Analyses using all available categories and longitudinal analyses using categorical SES are presented in the supplementary file. The base model was adjusted for age, sex ethnicity, and MAP. Models were then further adjusted for BMI, smoking status, and alcohol intake; SBP, heart rate, total cholesterol, high-density lipoprotein cholesterol, diabetes, and antihypertensive use; and all of the foregoing, following the approach adopted in a recent meta-analysis examining the independent effect of PWV on cardiovascular risk [9]. Changes in lifestyle risk factors and cardiovascular indicators between 2008–2009 and 2012–2013 were accounted for using time-dependent variables. We examined whether sex and age modified the association between SES, education, and PWV change by fitting three-way interactions between these variables, the SII, and time since baseline and found no statistically significant interactions. We used data from the aforementioned meta-analysis [9], to estimate the effect of the differential increases in PWV between low and high SES individuals on cardiovascular risk. Analyses were performed using SAS version 9.4 [23].

The Whitehall II study was reviewed and approved by the University College London Ethics Committee (85/0938). Written informed consent was obtained from each participant at each phase. The study was conducted according to the principles of the Helsinki Declaration.

## RESULTS

Table 1 presents the characteristics of the 5242 participants. The study sample was predominantly composed of men (73.2%). The mean age was 65.5 years old (SD = 5.8). Participants were mainly white (92.1%), nonsmokers (93.3%) and most have consumed alcohol in the past week (85.2%).

Table 2 and S1 present results from the cross-sectional analyses examining the association between education, SES, education, and baseline PWV. Low father's social class was associated with higher baseline PWV. The cross-sectional associations with employment grade, income, and education were not statistically significant.

**FIGURE 1:**
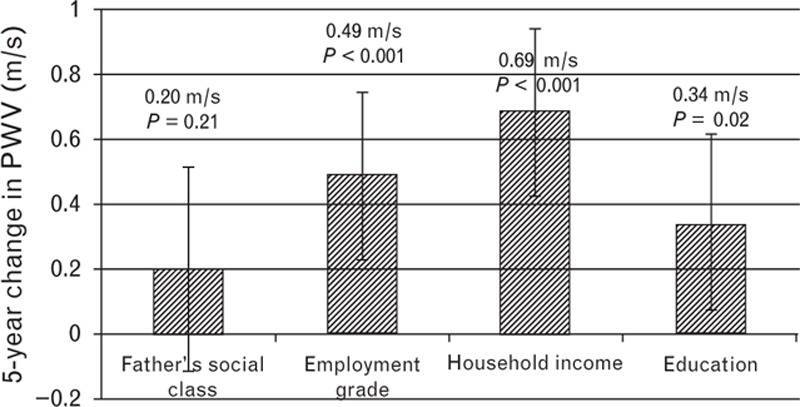
Slope index of inequality for 5-year change in aortic pulse wave velocity. Slope index comparing the lowest SES with the highest SES adjusted for age, sex, ethnicity, and mean arterial pressure. Bars show 95% confidence intervals. SES, socioeconomic status.

Figure 1 and Table S2 present the longitudinal associations between SES, education, and 5-year change in PWV, adjusting for age, sex, ethnicity, and MAP. As shown in Fig. 1, being in the lowest level of employment grade, household income and education was associated with greater increases in PWV. Adjusting for lifestyle-related and cardiovascular risk factors only slightly reduced these associations (Table 3). Moving from the highest to the lowest level of employment grade (0.38 m/s, 0.11, 0.65), household income (0.58 m/s, 0.32, 0.85) and education (0.30 m/s, 0.01, 0.58) was associated with higher absolute PWV increases, in the fully adjusted models. Based on Ben-Shlomo's meta-analytic estimates [9], the higher progression of PWV observed for participants with low household income would translate in a 6% increase in CVD risk.

## DISCUSSION

The present longitudinal study, conducted among a large sample of men and women, showed that lower educational attainment and adult SES are associated with more rapid progression of aortic stiffening. These associations were observed for household income, employment grade, and educational attainment, but not childhood circumstances, as assessed by father's social class. The associations were largely robust to adjustment for demographics, lifestyle-related risk factors, and other cardiovascular indicators. In the cross-sectional analysis, father's social class was associated with baseline PWV.

Household income was the SES indicator most strongly associated with PWV progression. Low family income was previously found to be associated with higher PWV among a small sample of American adolescents [10]. Income represents the flow of economic resources available to an individual [24]. Persons with lower income are more likely to have fewer resources to afford a variety of material needs such as safe housing, good nutrition, and health services [25]. Low income therefore relates directly to poor material living conditions that may affect cardiovascular health. The effect of household income could also partly be explained by the fact that low-income individuals had lower employment grade. However, the correlation between employment grade and household income was moderate (*r* = 0.56). Our findings suggest that household income cover additional dimensions of socioeconomic circumstances as supported by the stronger observed effect. According to our results, the economic dimension of SES could be of particular importance to explain social disparities in aortic stiffening at older ages.

The magnitude of the social gradient in PWV might also be sensitive to the proximal/distal nature of the employed socioeconomic indicator [26]. A measurement of SES closer in time to a health outcome may show stronger associations as they capture the current and accumulated socioeconomic circumstances of the individual more accurately. In the present study, income was measured at the time of the first PWV measurement, in 2008–2009. This contemporaneous assessment might be more suitable to assess the cumulative effect of social disadvantage through a person's life on PWV. We estimated that the higher 5-year progression of PWV observed for participants with low household income would translate in a 6% increase in CVD risk. Differences in PWV progression might be of higher magnitude over the whole lifespan. The effect of low SES-induced PWV increases on cardiovascular risk could therefore be larger. A third measurement of PWV is currently being collected in the present cohort and will allow us to clarify this hypothesis.

In the present study, employment grade was also associated with PWV progression. Studies have established a consistent relation between employment grade and CVD [27,28]. This relationship has also been observed at older ages (≥65) [29,30], consistent with our results. One possible explanation for the effect of employment grade on CVD and PWV lies in the fact that it is closely linked with characteristics of the work environment, such as work stress [31]. A number of prospective studies have documented the effect work stress on CVD incidence and recurrence [32,33]. Exposure to job strain was associated with higher PWV [34,35] in two cross-sectional studies conducted among Japanese workers. Further research is needed to clarify the role of the work environment in explaining the social gradient in PWV.

Low educational attainment was also robustly associated with PWV increases despite the weaker magnitude of the observed effect. The effect of education on PWV was previously reported in two previous cross-sectional studies [10,11]. Education is considered to be the indicator most likely to capture aspects of lifestyle and behaviors [24]. Results from the present study suggest that the association between education and PWV is largely robust to these risk factors, as we adjusted for alcohol intake, smoking, and BMI and change in these factors over the follow-up period. Nonetheless, other behavioral risk factors, including dietary intake have been shown to explain the inverse gradient between education and cardiovascular mortality and could contribute to explain the observed associations [36].

Socioeconomic disparities in aortic stiffness progression might also be mediated through biological pathways. For example, low employment grade was previously linked with coronary artery calcification [37]. Structural alterations in the vascular media, including calcification, are associated with increased PWV [38] and could therefore act as intermediate endpoints between low employment grade and PWV. In addition, low education attainment was found to be associated with inflammatory markers [39], which in turn were found to be associated with measures of arterial stiffness and wave reflection [40].

In the present study, father's social class was found to be associated with baseline PWV but not with PWV change over 5 years. This result suggests that alteration to arteries attributable to adverse social conditions in childhood might have already occurred at an earlier stage of life. This hypothesis is supported by Thurston and colleagues [10], who demonstrated that parental SES is associated with higher PWV in the adolescence. The long-lasting effect of those early alterations might partly explain the association found in the literature between childhood SES and cardiovascular morbidity and mortality [18].

There is a debate about the optimal model parameterization for modelling change. High baseline PWV may be the consequence of faster increases before the study's baseline measurement in low SES individuals. This effect, described as the horse-racing effect, is most likely to occur in observational cohort studies such as ours [41]. In this likely situation, baseline-adjusted models could lead to underestimations of the true effect of SES on PWV change over time. The retained strategy, where baseline PWV is considered as one of the outcome measures, is more likely to provide unbiased estimates [42].

Our study has limitations. First, there was considerable missing data on father's social class (32%) and education level (13%). We conducted a supplementary analysis comparing those who had missing values on those indicators with those who participated in the study, based on demographics and other socioeconomic variables (Tables S3 and S4 in the supplementary file). These analyses suggest that missingness was related to nonwhite ethnicity and low SES. We can therefore not exclude the possibility of selection bias, which underestimates effects of education and SES. Second, the Whitehall II cohort is composed of relatively healthy participants and does not include blue-collar workers, limiting generalizability of our findings. However, the cohort covers a wide socioeconomic range, as shown by the distribution of the sample in each SES indicators categories.

Our study has important strengths. It was conducted among a large sample of men and women. Aortic stiffness was measured using the same gold standard tonometry method at baseline and follow-up, using a rigorous protocol. Moreover, multiple indicators of SES were examined, each of them showing different strength of relationship with PWV. Finally, a large number of covariates have been considered, including demographics, lifestyle-related risk factors and cardiovascular indicators, which support the robustness of social disparities in aortic stiffening.

Aortic stiffness is an independent risk factor for hypertension, CVD, and stroke [9,43]. Primary CVD prevention strategies might benefit from early identification of individuals with fast progression of subclinical disease and at higher risk for cardiovascular events [44]. Multiple assessments of aortic PWV may prove to be a valuable tool to achieve that goal. The robust association found between adult SES and aortic stiffening in the present study supports the clinical relevance of examining the vascular ageing process in socioeconomically disadvantaged individuals.

The present longitudinal study supports the presence of socioeconomic disparities in aortic stiffness progression at older ages. Our findings suggest that arterial aging could be an important pathophysiological pathway explaining the impact of SES on CVD risk.

## ACKNOWLEDGEMENTS

Part of the work from this article has been previously presented at the 2015 Canadian Hypertension Congress and at the 2016 scientific meeting of the Quebec Hypertension Society.

The work was supported by a fellowship from the Canadian Institutes of Health Research, the British Heart Foundation (RG/13/2/30098), British Medical Research Council (K013351), the British Health and Safety Executive, the British Department of Health, the British Stroke Association (TSA 2008/05), the US National Heart, Lung, and Blood Institute (R01HL036310), and the US National Institute on Aging (R01AG013196 and R01AG034454).

### Conflicts of interest

There are no conflicts of interest.

## Supplementary Material

Supplemental Digital Content

## Figures and Tables

**TABLE 1:** Characteristics of the study participants (*N* = 5242)

Characteristics	*N* (%) or mean (SD)
Sex	
Male	3836 (73.2)
Female	1406 (26.8)
Age, 2008–2009 (year)	65.5 (5.8)
Ethnic group	
White	4825 (92.1)
Nonwhite	417 (7.9)
BMI, 2008–2009 (kg/m^2^)	26.4 (4.1)
Smoking status, 2008–2009	
No	4810 (93.3)
Yes	346 (6.7)
Alcohol intake in the past week, 2008–2009	
No	737 (14.8)
Yes	4234 (85.2)

Missing values were 5% or less for all covariates. SES, socioeconomic status.

**TABLE 2:** Socioeconomic status, education and pulse wave velocity at baseline (2008–2009)

	*N*	PWV at baseline (m/s) difference^a^ (95% CI) *P*
Father's social class		
* *I–II	1538	Ref (8.5)^b^
* *IIIn–IIIm	1698	+0.15 (0.005, 0.30) 0.04
* *IV–V	340	+0.21 (−0.05, 0.46) 0.11
Slope index of inequality^c^		0.25 (0.001, 0.50) 0.049
Employment grade		
* *Administrative	2571	Ref (8.6)^b^
* *Professional/executive	2182	+0.02 (−0.10, 0.15) 0.70
* *Clerical/support	467	+0.13 (−0.10, 0.36) 0.25
Slope index of inequality^c^		0.08 (−0.14, 0.29) 0.49
Household income		
* *£50 – >100 000	1366	Ref (8.6)^b^
* *£25 – 49 999	2084	+0.05 (−0.10, 0.20) 0.54
* *<£9999–24 999	1585	+0.11 (−0.06, 0.28) 0.20
Slope index of inequality^c^		0.19 (−0.03, 0.41) 0.09
Education		
* *BA/BSc and higher degree	1757	Ref (8.6)^b^
* *Advanced level	1295	+0.05 (−0.10, 0.20) 0.53
* *No academic/ordinary level	1494	+0.10 (−0.05, 0.25) 0.20
Slope index of inequality^c^		0.13 (−0.10, 0.36) 0.27

^a^Adjusted for age, sex, ethnicity, and mean arterial pressure.

^b^Adjusted mean level of pulse wave velocity in the reference category of each of the SES indicators. Estimates for each consecutive category represent the difference in adjusted mean PWV when compared with the reference level.

^c^Slope index of inequality comparing the lowest SES with the highest SES. CI, confidence interval; PWV, pulse wave velocity; SES, socioeconomic status.

**TABLE 3:** Slope index of inequality^a^ for 5-year change in aortic pulse wave velocity: sequential adjustment

		Change in pulse wave velocity (per 5 years)	
SES indicators	Model adjustments	Increase (95% CI)	*P* value
Father's social class	Base + lifestyle-related risk factors^b^	0.23 (−0.09, 0.55)	0.16
	Base + cardiovascular indicators^c^	0.14 (−0.18, 0.45)	0.39
	Base + all	0.16 (−0.16, 0.49)	0.33
Employment grade	Base + lifestyle-related risk factors^b^	0.46 (0.20, 0.72)	0.0006
	Base + cardiovascular indicators^c^	0.42 (0.17, 0.68)	0.0012
	Base + all	0.38 (0.11, 0.65)	0.005
Household income	Base + lifestyle-related risk factors^b^	0.66 (0.40, 0.92)	<0.001
	Base + cardiovascular indicators^c^	0.59 (0.34, 0.85)	<0.001
	Base + all	0.58 (0.32, 0.85)	<0.001
Education	Base + lifestyle-related risk factors^b^	0.35 (0.07, 0.63)	0.016
	Base + cardiovascular indicators^c^	0.29 (0.013, 0.56)	0.04
	Base + all	0.30 (0.01, 0.58)	0.04

^a^Slope index of inequality comparing the lowest SES with the highest SES. Base model is adjusted for age, sex, ethnic group, and mean arterial pressure at the time of the pulse wave velocity measurement.

^b^Lifestyle-related risk factors are BMI, smoking, and alcohol intake.

^c^Cardiovascular indicators are SBP, heart rate, total cholesterol, HDL cholesterol, diabetes, and antihypertensive use. HDL, high-density lipoprotein; SES, socioeconomic status.

## Reviewers’ Summary Evaluations

### Referee 1

The novelty of the study lies on the effect of the socioeconomic status (SES) on arterial stiffening over time measured by aortic pulse wave velocity (PWV). Additionally, the sample is very representative as long as it includes a large number of participants of both genders, which makes the study even more attractive. However, the association between SES and PWV is not novel since it has been previously reported. However, this study is complementary of these previous ones and highlights a strong association between socioeconomic factors and PWV as a subclinical CVD index.

### Referee 2

This study provides a novel assessment of the association between aortic stiffness progression and socioeconomic status. The authors propose a mechanism by which lower socioeconomic status may lead to increased risk of cardiovascular disease. The strengths of the paper are its novelty, robust methodology and the large prospective cohort. Appropriate indicators of socioeconomic status were used and confounders were considered. Although generalizability may be somewhat limited by disparities in gender and ethnicity, the findings are convincing in this cohort.
