# Supplementary material for: Socioeconomic status, education, and aortic stiffness progression over 5 years: the Whitehall II prospective cohort study
Source: J Hypertens. 2016 Sep 1;34(10):2038–44. doi: 10.1097/HJH.0000000000001057 (PMC5398902; doi:10.1097/HJH.0000000000001057)
Supplement: Supplemental Digital Content [file jhype-34-2038-s001.docx]

*SUPPEMENTARY ANALYSES*

Table S1. Mean difference in pulse wave velocity at baseline (2008-2009) according to each socio economic indicator (All categories)

|  | N | PWV at baseline (m/s)  Difference^1^ (95% CI) p |
| --- | --- | --- |
| **Father’s social class** |  |  |
| I | 382 | Ref (8.3) † |
| II | 1,156 | +0.02 (-0.18, 0.21) 0.86 |
| IIIn | 571 | +0.19 (-0.04, 0.41) 0.10 |
| IIIm | 1,127 | +0.19 (-0.003, 0.39) 0.054 |
| IV | 233 | +0.18 (-0.13, 0.49) 0.26 |
| V | 107 | +0.27 (-0.11, 0.66) 0.16 |
|  |  |  |
| **Education** |  |  |
| Higher Degree | 684 | Ref (8.6) † |
| BA/Bsc | 1,073 | -0.08 (-0.28, 0.12) 0.45 |
| A/S level | 1,295 | +0.001 (-0.20, 0.20) 0.99 |
| O level | 1,144 | +0.04 (-0.17, 0.24) 0.71 |
| No academic qualification | 350 | +0.10 (-0.20, 0.38) 0.51 |
|  |  |  |
| **Employment grade** |  |  |
| UG1-UG6 | 1,475 | Ref (8.6) † |
| UG7 | 1,096 | +0.06 (-0.11, 0.23) 0.47 |
| SEO | 794 | +0.03 (-0.15, 0.22) 0.73 |
| HEO | 770 | +0.05 (-0.13, 0.24) 0.57 |
| EO | 618 | +0.07 (-0.14, 0.28) 0.51 |
| Clerical/Support | 467 | +0.17 (-0.08, 0.41) 0.18 |
|  |  |  |
| **Household income** |  |  |
| £ >100, 000 | 261 | Ref (8.4) † |
| £ 70 – 99.999 | 412 | +0.18 (-0.15, 0.52) 0.28 |
| £ 50 – 69,999 | 693 | +0.15 (-0.16, 0.45) 0.35 |
| £ 35 – 49,999 | 1,030 | +0.20 (-0.10, 0.49) 0.18 |
| £25 – 34,999 | 1,054 | +0.16 (-0.14, 0.45) 0.29 |
| £20 – 24,999 | 630 | +0.08 (-0.23, 0.40) 0.61 |
| £15 – 19,999 | 419 | +0.21 (-0.12, 0.55) 0.21 |
| £10 – 14,999 | 387 | +0.43 (0.08, 0.78) 0.02 |
| <£9,999 | 149 | +0.68 (0.23, 1.13) 0.003 |

^1^ Adjusted for age, gender,ethnicity and mean arterial pressure

† Shows the adjusted mean level of pulse wave velocity in the reference category of each of the SES indicators. Estimates for each consecutive category represent the difference in adjusted mean PWV when compared to the reference level

Table S2. Socioeconomic status, education and 5-year change in aortic pulse wave velocity

|  | 5-year change in PWV (m/s)  Difference^1^ (95% CI) p |
| --- | --- |
| **Father’s social class** |  |
| I -II | Ref (0.49) † |
| IIIn – IIIm | 0.10 (-0.08, 0.28) 0.26 |
| IV-V | 0.20 (-0.12, 0.51) 0.22 |
|  |  |
| **Employment grade** |  |
| Adminstrative | Ref (0.43) † |
| Professional/Executive | 0.22 (0.07, 0.36) 0.004 |
| Clerical/ Support | 0.23 (-0.04, 0.50) 0.10 |
|  |  |
| **Household income** |  |
| £50 - >100, 000 | Ref (0.28) † |
| £25 – 49,999 | 0.30 (0.13, 0.48) 0.001 |
| <£9,999 – 24,999 | 0.46 (0.27, 0.65) <0.001 |
|  |  |
| **Education** |  |
| BA/BSc and Higher Degree | Ref (0.40) † |
| A/S level | 0.20 (0.02, 0.39) 0.03 |
| No academic / O level | 0.19 (0.01, 0.37) 0.04 |
|  |  |

^1^ Adjusted for age, gender, ethnicity and mean arterial pressure

† Shows the adjusted 5-year change in pulse wave velocity in the reference category of each of the SES indicators. Estimates for each consecutive category represent the difference in 5-year change when compared to the reference level

Table S3 Difference in Pulse wave velocity at baseline (2008-09) between participants at baseline and those excluded for missing data on SES variables

|  | N | PWV at baseline (m/s)  Difference ^1^ (95% CI) p |
| --- | --- | --- |
| **Father’s social class** |  |  |
| Participants | 3,061 | Ref (8.4) † |
| Missing | 1,286 | +0.06 (-0.05, 0.18) 0.28 |
| **Education** |  |  |
| Participants | 3,781 | Ref (8.5) † |
| Missing | 566 | +0.08 (-0.07, 0.24) 0.32 |
| **Employment grade** |  |  |
| Participants | 4,327 | Ref (8.5) † |
| Missing | 20 | -0.54 (-1.31, 0.23) 0.17 |
| **Household income** |  |  |
| Participants | 4,183 | Ref (8.5) † |
| Missing | 164 | 0.00 (-0.27, 0.28) 0.99 |

^1^ Adjusted for age, gender, ethnicity and mean arterial blood pressure

† Shows the adjusted mean level of pulse wave velocity among participants at baseline. Estimates for the missing category represent the difference in adjusted mean level of pulse wave velocity between participants and those excluded for missing data on SES variables.

|  | **Education** | | | **Father’s social class** | | |
| --- | --- | --- | --- | --- | --- | --- |
|  | Participants | Missing | p | Participants | Missing | p |
|  | N(%) | N(%) |  | N(%) | N(%) |  |
| **Age** (2008-09), y Mean SD | 65.3 (5.8) | 65.3 (5.6) | 0.84 | 65.2 (5.7) | 65.5 (5.8) | 0.18 |
| **Gender** |  |  |  |  |  |  |
| *Men* | 2,870 (75.9) | 367 (64.8) | > 0.001 | 2,301 (75.2) | 936 (72.8) | 0.10 |
| *Women* | 911 (24.1) | 199 (35.2) |  | 760 (24.8) | 350 (27.2) |  |
| **Ethnic group** |  |  |  |  |  |  |
| *White* | 3,513 (92.9) | 487 (86.0) | > 0.001 | 2,836 (92.7) | 1,164 (90.5) | 0.02 |
| *Non-white* | 268 (7.1) | 79 (14.0) |  | 225 (7.4) | 122 (9.5) |  |
| **Employment grade** |  |  |  |  |  |  |
| UG7 – UG1 | 1,944 (51.4) | 207 (37.9) | > 0.001 | 1,479 (48.6) | 672 (52.3) | 0.08 |
| EO/HEO/SEO | 1,557 (41.2) | 248 (45.4) |  | 1,298 (42.7) | 507 (39.5) |  |
| Clerical/ Support | 280 (7.4) | 91 (16.7) |  | 266 (8.7) | 105 (8.2) |  |
| **Household income** |  |  |  |  |  |  |
| £50 - >100, 000 | 1,015 (27.7) | 133 (25.8) | > 0.001 | 782 (26.5) | 366 (29.8) | 0.06 |
| £25 – 49,999 | 1,587 (43.3) | 169 (32.7) |  | 1,268 (42.9) | 488 (39.7) |  |
| <£9,999 – 24,999 | 1,065 (29.0) | 214 (41.5) |  | 903 (30.6) | 376 (30.6) |  |

Table S4 Difference in demographics and other SES indicators between participants at baseline and those excluded for missing data on education and father’s social class
